# An ecological study of the association between environmental indicators and early childhood caries

**DOI:** 10.1186/s13104-020-05321-w

**Published:** 2020-10-07

**Authors:** Morenike Oluwatoyin Folayan, Maha El Tantawi, Balgis Gaffar, Robert J. Schroth, Jorge L. Catillo, Ola B. Al-Batayneh, Arthur Kemoli, Aída Carolina Medina Díaz, Verica Pavlic, Maher Raswhan, for Early Childhood Caries Advocacy Group

**Affiliations:** 1 Department of Child Dental Health, Obafemi Awolowo University, Ile-Ife, Osun State Nigeria; 2 Department of Pediatric Dentistry and Dental Public Health, Faculty of Dentistry, Alexandria University, Alexandria, Egypt; 3 Department of Preventive Dental Sciences, College of Dentistry, Imam Abdulrahman Bin Faisal University, Dammam, Saudi Arabia; 4 Department of Preventive Dental Science, Dr. Gerald Niznick College of Dentistry, Rady Faculty of Health Sciences, University of Manitoba, Winnipeg, MB Canada; 5 Department of Dentistry for Children and Adolescents, Universidad Peruana Cayetano Heredia, Lima, Peru; 6 Preventive Dentistry Department, Jordan University of Science and Technology, PO Box 3030, Irbid, 22110 Jordan; 7 Department of Paediatric Dentistry and Orthodontics, University of Nairobi, Nairobi, Kenya; 8 Pediatric Dentistry and Orthodontics Department, Universidad Central de Venezuela, Caracas, Venezuela; 9 Department of Periodontology and Oral Medicine, Medical Faculty, University of Banja Luka, Banja Luka, Republic of Srpska Bosnia and Herzegovina; 10 Centre for Oral Bioengineering, Barts and the London, School of Medicine and Dentistry, Queen Mary University of London, Mile End Road, London, E1 4NS UK; 11 Department of Conservative Dentistry, Faculty of Dentistry, Alexandria University, Alexandria, Egypt

**Keywords:** Environmental performance indicators, Early childhood caries, Nitrous oxide emission, Methane emission

## Abstract

**Objectives:**

A prior study described the association between ecosystem vitality, environmental health, and early childhood caries (ECC). The objective of this study was to determine the association between 24 global environmental indicators and ECC in 3–5-year-old children.

**Results:**

In 61 countries, 55.5% of 3–5-year-old children had ECC. Eight factors had a small effect-size association with ECC: percentage of area that is marine-protected (partial eta squared; ƞ^2^ = 0.03); species habitat index (ƞ^2^ = 0.06); percentage of tree-cover loss (ƞ^2^ = 0.03); regional marine trophic index (ƞ^2^ = 0.03); total carbon dioxide emission intensity (ƞ^2^ = 0.03); methane emission intensity (ƞ^2^ = 0.04); nitrous oxide emission intensity (ƞ^2^ = 0.06); and sulfur dioxide emission intensity (ƞ^2^ = 0.03). Regression analysis revealed that two of these factors were significantly associated with the prevalence of ECC: methane emission intensity was inversely associated with ECC prevalence (B = − 0.34, 95% CI = − 0.66, − 0.03; p = 0.03), and nitrous oxide had a direct association with ECC prevalence (B = 0.35, 95% CI = 0.04, 0.67; p = 0.03).

## Introduction

Human health benefits from ecosystem biodiversity and from experiencing nature. Strong evidence links biodiversity, nature exposure, and human health [1]. Few empirical studies, however, have assessed possible links of the ecosystem and oral health. One such study investigated the association between ecosystem vitality, environmental health, and early childhood caries (ECC), which is any caries experience of the primary teeth in children < 72 months of age [2, 3]. The study identified an inverse and significant association between ECC in 3–5-year-olds and ecosystem vitality, and a direct but non-significant association between ECC prevalence and environmental health [3]. However, the study determined these associations by using composite ecosystem vitality and environmental health scores.

Environmental performance indicators include four that measure ecosystem vitality: (1) biodiversity and habitat (protected areas and species), (2) forests (tree cover loss), (3) fisheries (fish stocks), and (4) climate and energy (trend in carbon intensity and carbon dioxide emissions); and three indicators that measure environmental health: air quality, water and sanitation, and exposure to heavy metals.

A few publications have postulated a link between climate change and oral health, including ECC [6], but there are no empirical data on the link between the seven environmental performance indicators. Links have been suggested, though, between climate change, alterations in the concentration of greenhouse gases, and health problems [7]. The greenhouses gases (carbon dioxide, methane, water vapor, surface-level ozone, nitrous oxides, and fluorinated gases) absorb infrared radiation emitted from the earth’s surface and re-radiate it back to the earth’s surface, thereby warming the earth [8]. Global warming is associated with food shortages and the spread of diseases and pandemics, and it may aggravate cardiovascular and respiratory problems [9]. Food shortage can result in shortage of protein, energy, and micronutrients, which can lead to oral health problems, including caries [10]. Less is known about the direct impact of these gases on oral health.

Use of the environmental performance indicators is intended to capture the impact of various factors that interact to affect the health of the environment. A previous study [1] showed that the impact of some indicators seemed stronger and opposite in direction to other indicators. A closer look and more detailed analysis of the environmental indicators may shed light on how ECC and the seven indicators of environmental performance are associated. Therefore, this study aimed to investigate the association between the global indicators for environmental performance and ECC in 3–5-year-old children.

## Main text

### Methods

This was an ecological study that studied the on the association between ECC in 3–5-year-old children and the environment using environmental indicators [11]. The study covered the period from 2007 to 2017.

ECC data for children aged 3–5 years old were available for 85 United Nations States. These estimates were extracted from studies published between 2007 and June 2017 and indexed in MEDLINE, Web of Science, Scopus, and Google Scholar without language restriction. Nationally representative data were used to the greatest extent possible. Estimates from several studies were combined at the country level. The percentage of affected children was calculated as the number of children with ECC in all relevant studies divided by the total number of children examined and multiplied by 100 [11]. ECC was defined as the presence of one or more decayed, missing-due-to-caries, or cavitated non-cavitated carious tooth in a child younger than 6 years of age [2].

Environmental indicators data from the Yale Center for Environmental Law and Policy were used [4]. These data are ratings of the performance of countries on several environmental indicators arranged under two domains: environmental health (including three categories with six indicators) and ecosystem vitality (including seven categories with 18 indicators), resulting in a total of 24 indicators that describe the quality of the environment from various aspects. The values of the 24 indicators are reported at the country level on a score ranging from 0 to 100, with higher scores indicating better performance. The definitions of the indicators are presented in Additional file 1: Table S1. Data availability for different indicators varied, ranging from an indicator with data for 131 countries to indicators with data from 175 countries; 90 countries had complete data for the 24 indicators.

The data gathered were analyzed, and descriptive statistics (frequencies, percentages, means, and standard deviations) were calculated. Linear regression analysis was used to assess the association between the outcome variable (percentage of 3–5-year-old children with ECC) and the explanatory variables (environmental performance indicators), controlling for income level based on the gross national income (GNI). Countries were classified by GNI into low-income countries (GNI US$1025 or less), lower middle-income countries (GNI US$1026 to US$3995), upper middle-income countries (GNI US$3996 to US$12,475), and high-income countries (GNI US$12,476 or more [12].

Two sets of multivariable linear regression models were developed. For Model 1, each environmental performance indicator was entered one at a time with adjustment for income level. Indicators with partial eta squared (ƞ^2^) of at least small-effect size (ƞ^2^ = 0.02) [13] were entered into Model 2, where they were all mutually adjusted for in addition to the income level. Regression coefficients (B), 95% confidence intervals (CI), p values, and partial eta squared (ƞ^2^) were calculated. SPSS version 23.0 was used. Significance was set at 5%.

### Results

Combined ECC and environmental performance indicators data were available for 61 countries (Additional file 2: Table S2). Of these, two (3.3%) were low-income countries, 14 (23%) were low middle-income countries, 18 (29.5%) were upper middle-income countries, and 27 (44.3%) were high-income countries. The mean percentage of children with ECC was 55.5%. The mean percentage of environmental performance indicators ranged from a minimum of 22.3% for Tree Cover Loss to a maximum of 87.1% for Marine Protected Areas, as indicated in Table 1.

**Table 1:** Distribution environmental performance indicators and early childhood caries in 3–5-year-old children in the 61 countries included in the study

Factors	Percent score Mean (SD)
Early childhood caries prevalence	55.54 (22.14)
Household solid fuels	61.40 (32.23)
PM2.5 exposure	81.82 (25.61)
PM2.5 exceedance	82.63 (22.36)
Sanitation	65.27 (28.15)
Drinking water	64.55 (28.18)
Lead exposure	63.07 (23.91)
Marine protected areas	87.05 (14.49)
Terrestrial biome protection (National)	77.71 (27.00)
Terrestrial biome protection (Global)	76.93 (27.61)
Species protection index	79.56 (23.86)
Protected area representativeness index	48.34 (24.23)
Species habitat index	77.36 (18.40)
Tree cover loss	22.29 (20.07)
Fish stock status	60.64 (23.93)
Regional marine trophic index	50.94 (25.33)
CO_2_ emission intensity – total	47.96 (17.09)
CO_2_ emission intensity – power	46.87 (20.95)
Methane emission intensity	68.48 (19.44)
N_2_O emission intensity	57.71 (19.17)
Black carbon emission intensity	55.93 (21.32)
SO_2_ emission intensity	53.66 (25.18)
NOX emission intensity	50.91 (23.73)
Wastewater treatment	74.41 (30.20)
Sustainable nitrogen management index	38.03 (17.35)

Table 2 reports the association between individual environmental performance indicators and ECC in 3–5-year-old children adjusted for country income level in Model 1. Eight factors had at least a small-effect size association with ECC in 3–5-year-old children: percentage of marine protected areas (ƞ^2^ = 0.03), species habitat index (ƞ^2^ = 0.06), percentage of tree cover loss (ƞ^2^ = 0.03), regional marine trophic index (ƞ^2^ = 0.03), total carbon dioxide emission intensity (ƞ^2^ = 0.03), methane emission intensity (ƞ^2^ = 0.04), nitrous oxide emission intensity (ƞ^2^ = 0.06), and sulfur dioxide emission intensity (ƞ^2^ = 0.03).

**Table 2:** Association between ECC in 3–5-year-old children and environmental performance indicators controlling for income level

Indicators	Model 1			Model 2		
	B (95% CI)	P value	ƞ^2^	B (95% CI)	P value	ƞ^2^
Environmental health indicators						
Household solid fuels	− 0.05 (− 0.32, 0.23)	0.74	0.002			
PM2.5 exposure	− 0.02 (− 0.23, 0.19)	0.86	0.001			
PM2.5 exceedance	− 0.02 (− 0.26, 0.23)	0.88	< 0.0001			
Sanitation	0.01 (− 0.37, 0.39)	0.96	< 0.0001			
Drinking water	− 0.14 (− 0.52, 0.24)	0.46	0.01			
Lead exposure	− 0.01 (− 0.33, 0.31)	0.95	< 0.0001			
Ecosystem vitality indicators						
Marine protected areas	− 0.23 (− 0.60, 0.14)	0.22	0.03	− 0.05 (− 0.43, 0.33)	0.8	0.001
National terrestrial biome protection	0.003 (− 0.20, 0.21)	0.98	< 0.0001			
Global terrestrial biome protection	− 0.01 (− 0.22, 0.19)	0.9	< 0.0001			
Species protection index	− 0.10 (− 0.34, 0.14)	0.42	0.01			
Protected area representativeness index	− 0.10 (− 0.33, 0.13)	0.39	0.01			
Species habitat index	− 0.28 (− 0.56, 0.006)	0.06	0.06	− 0.15 (− 0.48, 0.19)	0.39	0.02
Tree cover loss	− 0.16 (− 0.42, 0.10)	0.23	0.03	− 0.12 (− 0.41, 0.18)	0.43	0.01
Fish stock status	− 0.06 (− 0.28, 0.16)	0.6	0.005			
Regional marine trophic index	0.13 (− 0.07, 0.33)	0.2	0.03	0.09 (− 0.11, 0.29)	0.39	0.02
Total CO_2_ emission intensity	− 0.22 (− 0.52, − 0.09)	0.16	0.03	− 0.07 (− 0.42, 0.28)	0.7	0.003
CO_2_ Emission intensity – power	− 0.10 (− 0.35, 0.15)	0.41	0.01			
Methane emission intensity	− 0.20 (− 0.47, 0.08)	0.15	0.04	− 0.34 (− 0.66, − 0.03)	0.03	0.09
N_2_O emission intensity	0.24 (− 0.03, 0.51)	0.07	0.06	0.35 (0.04, 0.67)	0.03	0.09
Black carbon emission Iiintensity	− 0.06 (− 0.34, 0.21)	0.64	0.004			
SO_2_ emission intensity	− 0.15 (− 0.36, 0.07)	0.18	0.03	− 0.04 (− 0.29, 0.20)	0.71	0.003
NOX emission intensity	− 0.04 (− 0.26, 0.18)	0.72	0.002			
Wastewater treatment	− 0.005 (− 0.25, 0.24)	0.97	< 0.0001			
Sustainable nitrogen management index	− 0.09 (− 0.42, 0.24)	0.6	0.005			

Model 1: including individual environmental performance indicators, one at a time, adjusted for income level; Model 2: including all environmental performance indicators, adjusted for income level; B: regression estimate, CI: confidence interval, ƞ^2^ = partial eta squared

These eight indicators were entered into Model 2, where they were mutually adjusted in addition to adjusting for income level. Model 2 explained 31% of the variation in the percentage of 3–5-year-old children with ECC. The model revealed that four factors had an association with the prevalence of ECC of at least small-effect size: species habitat index (ƞ^2^ = 0.02), regional marine trophic index (ƞ^2^ = 0.02), methane emission intensity (ƞ^2^ = 0.09), and nitrous oxide emission intensity (ƞ^2^ = 0.09).

Two indicators (species habitat index and methane emission intensity) were inversely associated with the prevalence of ECC. Higher species habitat index was non-significantly associated with lower percentage of children affected with ECC (B = − 0.15, 95% CI = − 0.48, 0.19; p = 0.39). Greater methane emission intensity was significantly associated with lower percentage of ECC-affected children (B = − 0.34, 95% CI = − 0.66, − 0.03; p = 0.03).

Two indicators (regional marine trophic index and nitrous oxide emission intensity) were directly associated with the prevalence of ECC. Higher regional marine trophic index was non-significantly associated with greater percentage of ECC-affected children (B = 0.09, 95% CI = − 0.11, 0.29; p = 0.39). Greater nitrous oxide emission intensity was associated with significantly higher percentage of 3–5-year-old ECC-affected children (B = 0.35, 95% CI = 0.04, 0.67; p = 0.03).

### Discussion

Our analysis revealed that methane and nitrous oxide emission intensities were risk indicators for ECC in 3–5-year-old children, though the associations were in opposite directions: methane emissions were associated with lower prevalence of ECC, and nitrous oxide emissions were associated with higher prevalence of ECC. This study provides evidence that some greenhouse gases also may be associated with poor oral health and are risk indicators for ECC in 3–5-year-old children, although with small effect.

Methane and nitrous oxide are two of the three most important long-lived greenhouse gases that contribute to global warming – the third being carbon dioxide [14]. Greenhouse gases are reported to have direct effects on public health, such as by causing heat-related morbidity and mortality and increasing the risk for vector-borne and food-borne diseases [15], with negative implications for the general health and wellbeing of children [16]. Perhaps knowing the relationships between indicators of environmental performance and ECC will foster more holistic approaches to improving children’s oral health.

Methane emission levels are generally higher in developing countries than in developed countries [17]. Sources of methane are mainly human activities related to animal agriculture and rice production [18–21], which account for 53% of the total anthropogenic methane emissions [22]. Higher methane emissions are frequently found in agricultural settings, where the diet is likely comprised of more locally sourced fruits, vegetables, grains, and meats and comprised less of refined carbohydrates. This dietary lifestyle would contribute to a lower prevalence of ECC [23] if ECC is caused indirectly by the surrounding agricultural environment and the lower socioeconomic status of people living in these agricultural areas rather than to a direct effect of this gas. However, a direct effect also is plausible: Methane has anti-inflammatory, anti-apoptosis, and antioxidant properties through which it exerts protective biological and clinical effects [24], one of which may be reducing the risk for caries, as the findings of this study suggest.

On the other hand, nitrous oxide is emitted through land-use change and fertilizer production [25] and from industry [26]; the dominant sources are closely related to microbial-production processes in soils, sediments, and water bodies [27]. Emission levels of the gas are higher in developed countries than in developing countries [28]. Poor regulation of nitrous oxide in industrialized urban environments may be a factor accounting for an association between nitrous oxide emission levels and ECC [3].

The pathophysiology of how these gases affect the risk for caries is not known. We postulate, though, that the absorption of gases present in the environment by the oral microbiome is the pathway to ECC, as methane has a neutral pH [29], and nitrous oxide is a neutral oxide [30]. However, the oral environment’s rich microbiome has high alkali-generating potential (urea and arginine to ammonia) that also can produce methane. The high alkali-methane rich oral environment may protect against caries [31]. A methane-rich environment may enhance oral absorption from the atmosphere [24], resulting in protective oral effects against caries. The pathway to increased caries risk in environments with high nitrous oxide emission levels is less clear. Caries requires the acidification of dental plaque that favors the emergence of an acidogenic and acid-tolerant (aciduric) microflora that promote caries formation [32]. Although nitrous oxide accumulates faster in oral cavities that have heavy accumulations of plaque [33], this does not explain how nitrous oxide in the environment is associated with the risk for ECC. Future studies will be needed to explain how these environmental indicators, measured at smaller administrative units, may promote or prevent the development of ECC. The results of such studies may enable control for various potential confounders, thereby leading to more robust conclusions about ECC-environment relationships.

## Limitations

This study has limitations. It is an ecological study that may have fallacies resulting from the use of multiple aggregated data sets. We acknowledge that in ecological studies, correlations tend to be larger when associations are determined at the group level rather than at the individual level [29]. The data also are skewed towards high-income countries and upper middle-income countries, which may limit generalizability of the findings. The study also is cross-sectional, so inferences on causal relationships cannot be made.

## Supplementary information

**Additional file 1: Table S1.** Definition of environmental indicators.

**Additional file 2: Table S2.** Countries included in the study and their indicators.

## Data Availability

Study related materials are public data. All study related data are included in the supplemental file of this manuscript.

## Abbreviations

B: Regression coefficient
CI: Confidence interval
ECC: Early Childhood Caries
EPI: Environmental performance index
GNI: Gross national income
HICs: High income countries
LICs: Low income countries
LMICs: Low middle income countries
ƞ^2^: Partial eta squared
UMICs: Upper middle-income countries
