# Supplementary material for: An ecological study of the association between environmental indicators and early childhood caries
Source: BMC Res Notes. 2020 Oct 7;13:474. doi: 10.1186/s13104-020-05321-w (PMC7542688; doi:10.1186/s13104-020-05321-w)
Supplement: Supplementary file 1 [file 13104_2020_5321_MOESM1_ESM.odt]

Table S1: Definition of environmental indicators

| Abbreviation | Full name | Definition | Best | Worst |
| --- | --- | --- | --- | --- |
| HAD | Household Solid Fuels | Measures the actual outcomes from exposure to indoor air pollution from household use of solid fuels in age-standardized Disability-Adjusted Life Years (DALY) lost per 100,000 persons, or the DALY rate | 5th percentile | 95th percentile |
| PME | PM2.5 Exposure | Measures the average annual concentration of PM2.5 to which the typical citizen of each country is exposed in μg/m3 | 10 μg/m3 | 99th percentile |
| PMW | PM2.5 Exceedance | Measures the weighted percentage of a country’s population exposed to annual concentrations of PM2.5 that exceed WHO guidelines at four different levels: 10, 15, 25, and 35 μg/m3. Higher concentration levels are given higher weights in the averaging process in % of population | 0 | 99th percentile |
| USD | Sanitation | Measures the actual outcomes from lack of access or use of improved sources of drinking water in age-standardized DALY lost per 100,000 persons, or the DALY rate | 5th percentile | 95th percentile |
| UWD | Drinking Water | Measures the actual outcomes from lack of access or use of improved sources of drinking water in age-standardized DALY lost per 100,000 persons, or the DALY rate | 5th percentile | 95th percentile |
| PBD | Lead Exposure | Measures the actual outcomes from lead exposure in Age- standardized DALY lost per 100,000 persons, or the DALY rate | 1st-percentile | 99th percentile |
| MPA | Marine Protected Areas | Measures the percent of a country’s Economic Exclusion Zone (EEZ) set aside as a marine protected area (MPA) in % of EEZ | 10% | 0% |
| TBN | Terrestrial Biome Protection (National) | Measures the percent of a country’s biomes in terrestrial protected areas (TPAs), weighted by the prevalence of different biome types within that country in % of EEZ | 17% | 0% |
| TBG | Terrestrial Biome Protection (Global) | Measures the percent of a country’s biomes in terrestrial protected areas (TPAs) weighted by the prevalence of different biome types around the world in % of EEZ | 17% | 0% |
| SPI | Species Protection Index | Measures protected areas in relation to species distributions. The proportion of a species range in a country under protection is calculated for each species as Area of species range in country protected/Area of species range in country and capped at a maximum of 0.17. This value is then averaged for all species occurring in a country, with all species weighted equally in % of habitat | 17% | 0% |
| PAR | Protected Area Representativeness Index | Measures the extent to which a country’s protected areas are ecologically representative. It is a unitless index | 95th percentile | 5th percentile |
| SHI | Species Habitat Index | Measures changes in the suitable habitats of species to provide aggregate estimates of potential population losses and extinction risk increases. Each species is assessed separately, and the index is calculated as a weighted average of the habitat changes for each species with weights determined by the proportion of global range found in the country in % of habitat | 100 | 1st percentile |
| TCL | Tree Cover Loss | Measures the five-year moving average of percent of forests lost. Forests are defined as land areas having ≥30% canopy cover. Area of forested land (FOR) represents the forested land area at ≥30% canopy cover in the year 2000 in % of forested land | 5th percentile | 95th percentile |
| FSS | Fish Stock Status | Measures the percentage of a country’s total catch that come from taxa that are classified as either over-exploited or collapsed in % of catch. | 0 | 99th percentile |
| MTR | Regional Marine Trophic Index | Measures the trends in the Regional Marine Trophic Indices of a country, or mean trophic level of the fish catch in each region of the country’s Economic Exclusion Zones (EEZs). It is a unitless index. | 95th percentile | 5th percentile |
| DCT | CO2 Emission intensity – Total | Measures the intensity of CO2 emissions from the entire economy, as a blend of current-year intensity and a 10-year trend. It is unitless | 95th percentile | 5th percentile |
| DPT | CO2 Emission intensity – Power | Measures the intensity of CO2 emissions per kilowatt-hour of electricity and heat, as a blend of current-year intensity and a 10-year trend. It is unitless | 95th percentile | 5th percentile |
| DMT | Methane Emission Intensity | Measures the intensity of methane emissions from the entire economy, as a blend of current-year intensity and a 10-year trend. It is unitless | 95th percentile | 5th percentile |
| DNT | N2O Emission Intensity | Measures the intensity of N2O emissions from the entire economy, as a blend of current-year intensity and a 10-year trend. It is unitless | 95th percentile | 5th percentile |
| DBT | Black Carbon Emission Intensity | Measures the intensity of Black Carbon emissions from the entire economy, as a blend of current-year intensity and a 10-year trend. It is unitless | 95th percentile | 5th percentile |
| DST | SO2 Emission Intensity | Measures the intensity of SO2 emissions from the entire economy, as a blend of current-year intensity and a 10-year trend. It is unitless | 95th percentile | 5th percentile |
| DXT | NOX Emission Intensity | Measures the intensity of NOX emissions from the entire economy, as a blend of current-year intensity and a 10-year trend. It is unitless | 95th percentile | 5th percentile |
| WWT | Wastewater Treatment | Measures the percentage of wastewater treated, weighted by the connection rate of the population to the wastewater treatment system in weighted %. | 100 | 0 |
| SNM | Sustainable Nitrogen Management Index | Measures the Euclidean distance from an ideal point with optimal nitrogen use efficiency (NUE) and crop yield. It is unitless | 0 | 99th percentile |
